# Invasive pulmonary aspergillosis as a sentinel manifestation of good syndrome in a patient with thymoma

**DOI:** 10.1016/j.idcr.2026.e02727

**Published:** 2026-08-19

**Authors:** Mahshid Movahedi, Zahra Arabi, Shahin farzadmanesh

**Affiliations:** a Division of Allergyand Clinical Immunology, Department of Pediatric, Children Medical Center, Tehran University of Medical Sciences, Tehran, Iran; b Division of Gastroenterology & Hepatology, Imam Khomeini Hospital Complex, Tehran University of Medical Sciences, Tehran, Iran; c Division of Allergy and Clinical Immunology, Department of Pediatric, Children's medical Center, Tehran University of Medical Sciences, Tehran, Iran

**Keywords:** Good syndrome, Invasive pulmonary aspergillosis, Thymoma, Hypogammaglobulinemia, Opportunistic fungal infection

## Abstract

**Background:**

Good syndrome is a rare adult-onset combined immunodeficiency associated with thymoma, characterized by hypogammaglobulinemia and impaired T-cell–mediated immunity. Opportunistic infections are a major cause of morbidity and mortality, and invasive aspergillosis may represent a severe but underrecognized manifestation. In some cases, it may serve as a sentinel presentation leading to the diagnosis of Good syndrome.

**Case presentation:**

A 52-year-old man presented with a history of thymoma and myasthenia gravis, chronic sinopulmonary infections, cytomegalovirus colitis, chronic diarrhea, and cytopenia. Chest CT scan revealed multiple pulmonary nodules and tree-in-bud opacities. Bronchoalveolar lavage, galactomannan assay, and lung biopsy confirmed Aspergillus infection. Subsequent comprehensive immunologic evaluation demonstrated hypogammaglobulinemia, markedly reduced peripheral B cells (CD19⁺), and impaired T-cell immunity, consistent with Good syndrome.

**Conclusion:**

Invasive pulmonary aspergillosis can serve as a key sentinel manifestation leading to the diagnosis of Good syndrome in patients with thymoma. Early recognition of opportunistic infections and prompt immunological assessment are critical for timely treatment and improved clinical outcomes.

## Highlights

• Invasive pulmonary aspergillosis can be a sentinel manifestation of Good syndrome.
• Good syndrome should be suspected in thymoma patients with opportunistic infections.
• Combined humoral and cellular defects predispose to severe recurrent infections.
• Early immunologic evaluation is essential in atypical fungal and viral infections.

## Introduction

Good syndrome (GS) is a rare thymoma-associated immunodeficiency first described by Robert A. Good in 1954. It is characterized by adult-onset hypogammaglobulinemia, absent or markedly reduced B cells, and impaired T-cell–mediated immunity [1]. Unlike other primary antibody deficiencies, GS is notable for its high frequency of opportunistic infections, including cytomegalovirus, Candida, and Aspergillus species [[2], [3], [4]].

Invasive aspergillosis is classically associated with neutropenia, hematologic malignancy, or solid organ transplantation; however, it may also occur in patients with combined immunodeficiency [[5], [6], [7]]. In GS, impaired cellular immunity and defective antibody responses create a permissive environment for invasive fungal disease. Recognition of aspergillosis in patients with thymoma is therefore critical, as it may serve as a sentinel manifestation leading to the diagnosis of GS [4].

Here, we report a case of Good syndrome in which recurrent IPA was the predominant and presenting clinical feature, highlighting the diagnostic challenge and the need for early immunologic evaluation in patients with thymoma and atypical fungal infections.

### Case presentation

A 52-year-old man was referred to Imam Khomeini Hospital Complex, Tehran, Iran, for evaluation of recurrent pulmonary infections, chronic diarrhea, transfusion-dependent anemia, and progressive weight loss of approximately 10 kg over the two months preceding presentation. He had been diagnosed with myasthenia gravis nine years earlier and had been treated with pyridostigmine, systemic corticosteroids, azathioprine, and plasmapheresis. During the evaluation for myasthenia gravis, chest computed tomography (CT) revealed an anterior mediastinal mass consistent with thymoma. The patient subsequently underwent complete thymectomy later that same year (2016), and histopathological examination confirmed the diagnosis of thymoma.

### Clinical course focused on aspergillus infection

Approximately 3 years before the current admission (January 2023), the patient developed a chronic productive cough with thick purulent sputum, progressive dyspnea, and chronic rhinosinusitis refractory to medical therapy. He subsequently underwent functional endoscopic sinus surgery (FESS). Owing to persistent respiratory symptoms and recurrent lower respiratory tract infections, an extensive diagnostic evaluation was performed to determine the underlying cause of his pulmonary disease.

High-resolution computed tomography (HRCT) of the chest demonstrated a right pulmonary nodular lesion accompanied by multiple centrilobular nodules with a tree-in-bud pattern, fibroatelectatic changes, and cavitary lesions, raising suspicion for malignancy, granulomatous disease, or invasive fungal infection.

Further investigations for systemic vasculitis and other active autoimmune diseases were unremarkable. In addition, a comprehensive evaluation for sarcoidosis, including HRCT findings and histopathological assessment, revealed no evidence of non-caseating granulomas, effectively excluding sarcoidosis.

Bronchoscopy revealed abundant thick mucoid secretions. Aspergillus PCR performed on bronchoalveolar lavage (BAL) fluid was positive, and the BAL galactomannan index was 3.8, whereas serum galactomannan was negative. Fungal culture of the BAL specimen grew Aspergillus fumigatus. Furthermore, CT-guided biopsy of the right pulmonary nodule demonstrated Aspergillus hyphae without evidence of malignancy or vasculitis. Collectively, these microbiological, radiological, and histopathological findings established the diagnosis of invasive pulmonary aspergillosis (IPA), and antifungal therapy with voriconazole was initiated (Table 1).

**Table 1:** Summary of infectious manifestations and microbiologic findings.

**Site of Infection**	**Pathogen Identified**	**Diagnostic Method**	**Treatment**
Lung (parenchyma)	Aspergillus spp.	BAL culture, BAL galactomannan, lung biopsy	Voriconazole
Lung (airways)	Pseudomonas aeruginosa	Repeated sputum cultures	Cefepime, Ciprofloxacin
Gastrointestinal tract	Cytomegalovirus	Histopathology, PCR	Ganciclovir
Upper airway	Chronic bacterial sinusitis	Clinical, CT imaging	FESS antibiotics

FESS:(functional endoscopic sinus surgery) -PCR: Polymerase Chain Reaction- BAL: Bronchoalveolar lavage- spp: species

Despite antifungal treatment, the patient's clinical course was characterized by recurrent pulmonary exacerbations. Over the following months, repeated sputum cultures consistently yielded Pseudomonas aeruginosa despite ongoing voriconazole therapy, suggesting underlying structural lung disease and an associated immunodeficiency. The combination of recurrent respiratory infections, persistent opportunistic infection, and repeated isolation of P. aeruginosa heightened the suspicion of an underlying primary immunodeficiency, prompting a comprehensive immunological evaluation that ultimately led to the diagnosis of Good syndrome.

### Other systemic manifestations

At the time of admission to Imam Khomeini Hospital Complex, the patient presented not only with a history of recurrent pulmonary infections but also with progressive weight loss of approximately 10 kg over the preceding two months, chronic diarrhea, hematochezia, and severe transfusion-dependent anemia requiring monthly packed red blood cell (PRBC) transfusions.

Evaluation of the chronic diarrhea and gastrointestinal bleeding included upper and lower gastrointestinal endoscopy, which revealed erosive gastritis and cytopathological findings consistent with cytomegalovirus (CMV) colitis. CMV polymerase chain reaction (PCR) testing was positive, and intravenous ganciclovir therapy was initiated.

Bone marrow aspiration and biopsy, performed on two separate occasions, showed no evidence of hematologic malignancy, bone marrow failure, or myelodysplastic changes.

Laboratory investigations demonstrated elevated inflammatory markers (erythrocyte sedimentation rate [ESR], 52 mm/h; C-reactive protein [CRP], 28 mg/L), a positive antinuclear antibody (ANA) titer of 1:160, negative vasculitis screening, and negative serologic tests for human immunodeficiency virus (HIV) and hepatitis B and C viruses. Further evaluation revealed no evidence of other active autoimmune diseases or systemic vasculitis.

Given the patient's history of thymoma, invasive pulmonary aspergillosis, CMV colitis, and recurrent bacterial respiratory infections, an underlying combined immunodeficiency was strongly suspected. Comprehensive immunological evaluation demonstrated hypogammaglobulinemia, profound depletion of peripheral B lymphocytes, and impaired cellular immunity, findings consistent with the diagnosis of Good syndrome (Table 2).

**Table 2:** Immunologic profile supporting the diagnosis of good syndrome (based on patient data).

**Parameter**	**Patient Finding**	**Reference Range**	**Interpretation**
Serum IgG	640 mg/dL	700–1600 mg/dL	hypogammaglobulinemia consistent with GS
Serum IgA	295 mg/dL	70–400 mg/dL	Impaired mucosal humoral immunity
Serum IgM	18 mg/dL	40–230 mg/dL	Defective primary antibody response
Peripheral B cells (CD19⁺)	Severely reduced / absent	5–20% of lymphocytes	Hallmark feature of Good syndrome
CD4⁺ T cells	526.26 cells/µL	500–1500 cells/µL	Cellular immunodeficiency predisposing to opportunistic infections
CD8⁺ T cells	981.02 cells/µL	300–900 cells/µL	Leads to CD4/CD8 imbalance
CD4/CD8 ratio	0.6	> 1.0	Typical immunophenotype in GS

IgG: Immunoglobulin G -Ig A:Immunoglobulin A -IgM: Immunoglobulin M

### Chest CT findings

**Fig. 1:**
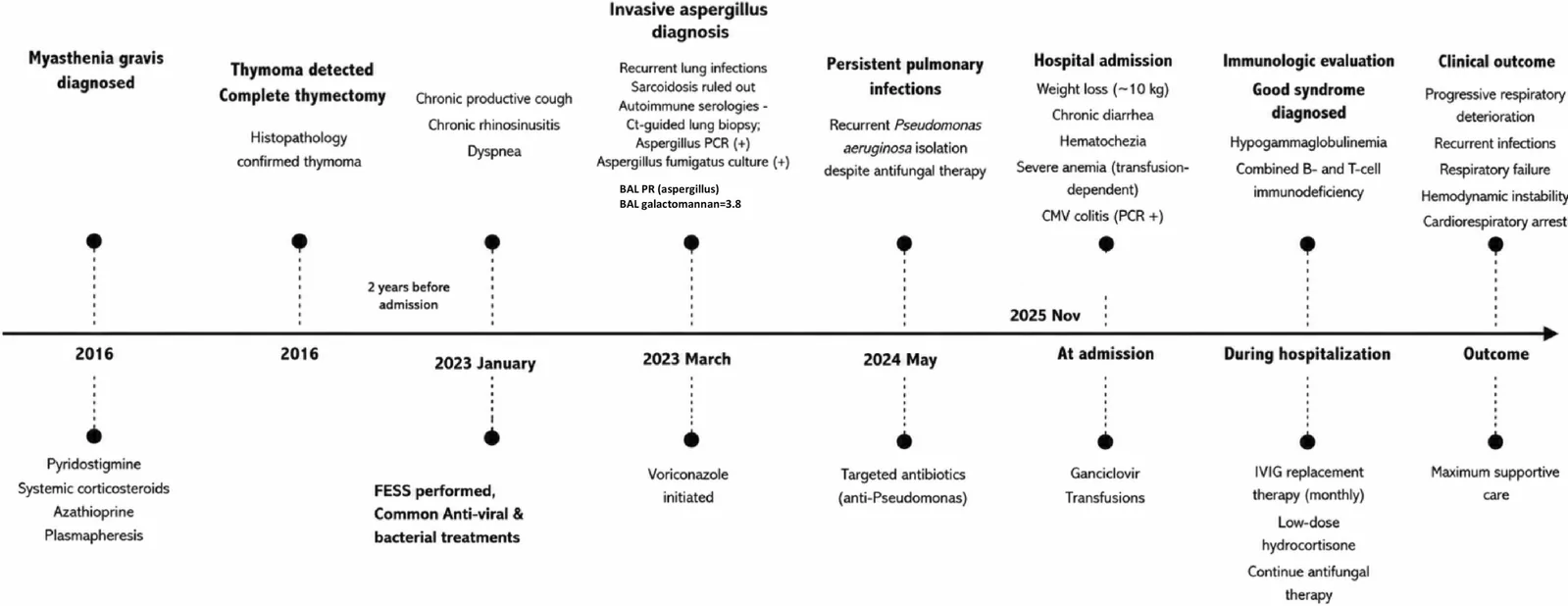
Non-contrast CT chest showing multiple centrilobular nodules with a tree-in-bud appearance, predominantly involving the bilateral lung bases.

Non-contrast spiral chest computed tomography (CT) demonstrated multiple bilateral centrilobular nodules with a tree-in-bud pattern, predominantly involving the lower lobes, consistent with infectious bronchiolitis or small-airway inflammation. Additional findings included fibrotic changes with associated right upper lobe atelectasis and multiple solid pulmonary nodules in the right lung, the largest measuring 8.5 mm in diameter (Fig. 1).

### Treatment and follow-up

Following the diagnosis of Good syndrome, immunoglobulin replacement therapy was initiated with intravenous immunoglobulin (IVIG) at a loading dose of 1 g/kg, followed by maintenance therapy at 500 mg/kg every four weeks. Hydrocortisone was continued at a dose of 10 mg each morning and 5 mg each evening. High-dose systemic corticosteroids were avoided because of the substantial risk of exacerbating the invasive fungal infection. Antifungal therapy with voriconazole was continued under close clinical and radiological monitoring.

**Fig. 2:**
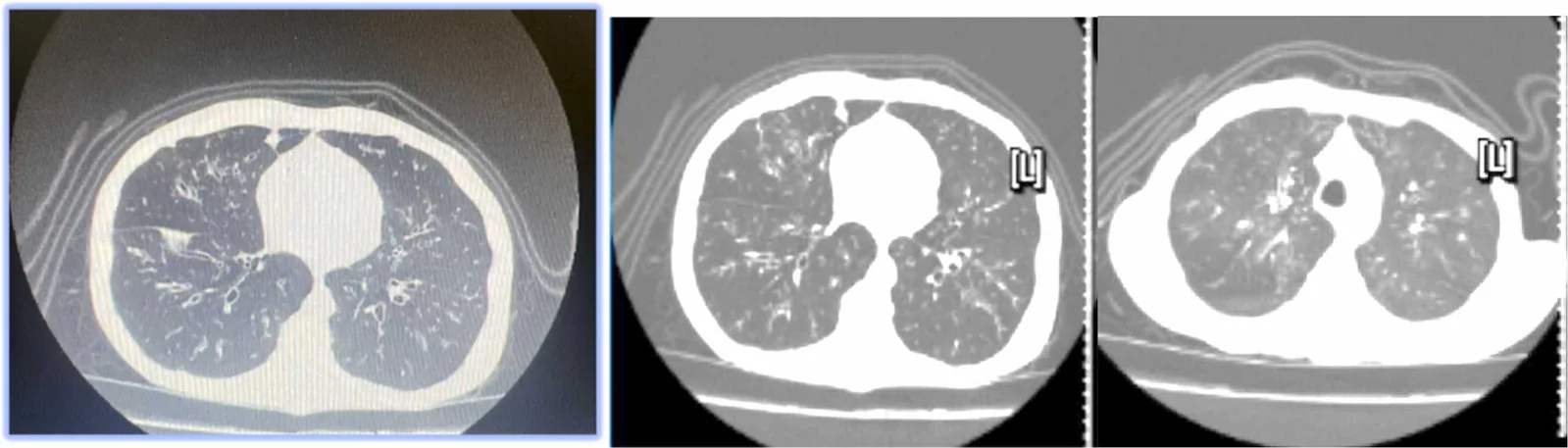
timeline.

Despite these interventions, the patient experienced progressive deterioration of pulmonary function with recurrent respiratory infections, reflecting the severity of the underlying immunodeficiency. Over time, his dyspnea and hypoxemia worsened progressively. Despite maximal supportive care and ongoing medical treatment, respiratory failure advanced, ultimately leading to hemodynamic instability followed by cardiopulmonary arrest. Cardiopulmonary resuscitation was unsuccessful, and the patient died.(Fig. 2)

## Discussion

This case underscores IPA as a major and potentially diagnostic manifestation of Good syndrome [8]. In contrast to isolated antibody deficiencies, GS involves combined defects in humoral and cellular immunity, predisposing patients to opportunistic fungal infections [9,10]. Aspergillus infection in patients with thymoma should prompt evaluation for GS, particularly when infections are recurrent, refractory, or invasive [9].

Radiologic findings such as mass-like lesions and cavitation may mimic malignancy, vasculitis, or granulomatous disease, frequently leading to diagnostic delay [11].In the present case, CT imaging was instrumental in directing further invasive diagnostics. Definitive diagnosis required integration of imaging, BAL galactomannan testing, and histopathologic confirmation.

Management of invasive aspergillosis in GS is challenging. Prolonged antifungal therapy is often required due to persistent immune dysfunction [12] According to current guidelines, voriconazole and isavuconazole are the preferred first-line agents for the treatment of pulmonary invasive aspergillosis, whereas liposomal amphotericin B is an alternative in selected patients; [13] however, relapse is common, particularly in the absence of immune reconstitution. Concomitant bacterial infections and viral reactivations, as seen in our patient, further complicate management. Avoidance of unnecessary immunosuppression and early initiation of IVIG are critical adjunctive strategies.

Notably, thymectomy does not reverse immunologic abnormalities in GS, and opportunistic infections may develop years after tumor resection [3]. Reported survival in GS remains limited, Mortality in Good syndrome is primarily driven by severe opportunistic infections, which remain the leading cause of death [10,14]. Early recognition of aspergillosis as a sentinel infection may therefore improve outcomes through earlier diagnosis and intervention.

## Conclusion

Invasive Pulmonary Aspergillus can represent both a sentinel and life-threatening manifestation of Good syndrome. Clinicians should maintain a high index of suspicion for GS in patients with thymoma who develop opportunistic fungal infections. Management remains challenging due to persistent combined immunodeficiency, even with optimal antifungal therapy, highlighting the importance of early immunologic evaluation, timely initiation of intravenous immunoglobulin (IVIG), and avoidance of unnecessary immunosuppression. Early recognition, comprehensive assessment, and coordinated use of immunoglobulin replacement alongside prolonged antifungal treatment are critical to improving patient outcomes.

## Abbreviations

IPA: Invasive Pulmonary Aspergillosis
GS: Good Syndrome

## Ethics declaration

Written informed consent to take part in the study and to publish the article has been obtained from all participants or their legal representatives. The privacy rights of participants have been observed.

This study was performed in compliance with relevant laws, regulatory frameworks and guidelines where the research took place. Ethics committee approval was not required under relevant laws and institutional guidelines. Ethics committee approval was not required for this case report in accordance with applicable institutional guidelines and regulations.

This research follows the CARE guidelines and the CARE checklist.

## Ethics Statement

Written informed consent was obtained from the patient for publication of this case report. The study was conducted in accordance with the principles of the Declaration of Helsinki.

## Funding

No specific funding was received for this work.

## CRediT authorship contribution statement

**Mahshid Movahedi:** Methodology, Supervision, Writing – original draft, Writing – review & editing. **Zahra Arabi:** Data curation, Resources, Validation, Writing – review & editing. **Shahin farzadmanesh:** Data curation, Methodology, Project administration, Validation, Visualization, Writing – review & editing.

## Declaration of Competing Interest

The authors declare that they have no known competing financial interests or personal relationships that could have appeared to influence the work reported in this paper
